# A Framework that Considers the Impacts of Time, Cost, and Uncertainty in the Determination of the Cost Effectiveness of Toxicity‐Testing Methodologies

**DOI:** 10.1111/risa.13810

**Published:** 2021-09-07

**Authors:** Paul S. Price, Bryan J. Hubbell, Shintaro Hagiwara, Greg M. Paoli, Daniel Krewski, Annette Guiseppi‐Elie, Maureen R. Gwinn, Norman L. Adkins, Russell S. Thomas

**Affiliations:** ^1^ Center for Computational Toxicology and Exposure US Environmental Protection Agency, Research Triangle Park Durham NC USA; ^2^ Air, Climate, and Energy Research Program US Environmental Protection Agency, Research Triangle Park Durham NC USA; ^3^ School of Mathematics and Statistics Carleton University Ottawa Canada; ^4^ Risk Sciences International Ottawa Canada; ^5^ McLaughlin Centre for Population Health Risk Assessment University of Ottawa Ottawa Canada; ^6^ Sustainable and Healthy Communities Research Program US Environmental Protection Agency, Research Triangle Park NC USA

**Keywords:** Cost effectiveness analysis, decision making, toxicity testing

## Abstract

Regulatory agencies are required to evaluate the impacts of thousands of chemicals. Toxicological tests currently used in such evaluations are time‐consuming and resource intensive; however, advances in toxicology and related fields are providing new testing methodologies that reduce the cost and time required for testing. The selection of a preferred methodology is challenging because the new methodologies vary in duration and cost, and the data they generate vary in the level of uncertainty. This article presents a framework for performing cost‐effectiveness analyses (CEAs) of toxicity tests that account for cost, duration, and uncertainty. This is achieved by using an output metric—the cost per correct regulatory decision—that reflects the three elements. The framework is demonstrated in two example CEAs, one for a simple decision of risk acceptability and a second, more complex decision, involving the selection of regulatory actions. Each example CEA evaluates five hypothetical toxicity‐testing methodologies which differ with respect to cost, time, and uncertainty. The results of the examples indicate that either a fivefold reduction in cost or duration can be a larger driver of the selection of an optimal toxicity‐testing methodology than a fivefold reduction in uncertainty. Uncertainty becomes of similar importance to cost and duration when decisionmakers are required to make more complex decisions that require the determination of small differences in risk predictions. The framework presented in this article may provide a useful basis for the identification of cost‐effective methods for toxicity testing of large numbers of chemicals.

## 1 INTRODUCTION

Currently, large numbers of chemical substances are created and used in a variety of commercial applications across the world's economies. A 2019 survey of 22 chemical inventories from 19 countries found that more than 137,000 unique organic chemicals and more than 9,300 unique inorganic chemicals were registered in one or more of these inventories (Wang, Walker, Muir, & Nagatani‐Yoshida, 2020). Evaluating the impacts of these chemicals on human health and the environment requires data on their toxicity. However, only a small fraction of these chemicals has been fully tested for potential toxicological effects (Judson et al., 2009) and an even smaller fraction has been evaluated in a formal risk assessment. The reason that governments do not test, or do not require full testing, for potential toxicological effects is primarily economic (Bottini & Hartung, 2009). For example, a full toxicological battery of tests for a pesticide can cost between $8 and $16 million U.S. dollars (Craig et al., 2019), while the time required for testing and chemical assessment can take eight years or longer (Krewski et al., 2020). Such costs and time requirements pose a major hinderance to innovation in chemical manufacturing and bringing consumer products containing new chemical to market.

The resource intensive nature of traditional toxicological testing—including both time and cost—and the lack of data for many chemicals has spawned a number of initiatives to improve toxicity testing by increasing speed, reducing costs, and improving efficiency. Many of these efforts have focused on replacing traditional *in vivo* test systems with a range of *in silico, in vitro*, and *in chemico* methods, along with integrated approaches to testing and assessment (CCA, 2012; Cronin et al., 2009; Krewski et al., 2010; Patlewicz et al., 2019; Thomas et al., 2019). Although progress has been slow, the release of strategic planning documents across multiple U.S. federal agencies (ICCVAM, 2018; U.S. FDA, 2017; U.S. EPA, 2018a, 2018b, 2020) has generated new momentum toward development and application of alternative toxicity‐testing methods in regulatory decisions. These renewed efforts will result in regulatory agencies having a choice of multiple test methods that could be used for evaluating health and environmental risks of untested chemicals. The selection of the most appropriate testing methodology requires balancing differences in the cost and the duration of testing and the measurement uncertainty in the resulting findings (hereafter referred to as uncertainty) across the suite of toxicity‐testing methodologies under consideration (Norlen, Worth, & Gabbert, 2014).

The cost of testing an individual chemical, the duration of the testing, and the uncertainty in the test results impact the usefulness of a testing methodology in different ways. Both higher costs and longer durations of testing reduce testing throughput. As budgets for testing are limited, greater testing costs decrease the number of chemicals that can be tested. Longer testing durations delay access to the benefits of testing. As discussed below, such benefits include health benefits from regulatory actions to reduce exposures for chemicals that pose risks and the ability to identify chemicals with low toxicity that can be safely used in commercial products. Standard economic theory recognizes that society prefers benefits occurring now rather than later. Thus, benefits realized in the future are inherently worth less today than benefits occurring now (Neumann, Sanders, Russell, Siegel, & Ganiats, 2016; U.S. EPA, 2010). The usefulness of toxicity data is a function of the ability of the data to enable optimal risk‐management decisions when identifying and controlling risks from chemical exposures. The uncertainty in toxicity data results in uncertainty in corresponding risk estimates. Risk management decisions based on such estimates may be less than optimal. As a result, uncertainty reduces the value of the toxicity data.

Cost effectiveness analysis (CEA) is a decision‐support tool that systematically evaluates different courses of action to determine the option that achieves a specified desirable outcome at the lowest cost. Unlike benefit‐cost analysis, it is not necessary to assign a monetary value to the benefits achieved in a CEA; however, outcomes are quantified and consistently defined across the various courses of action. CEAs place costs and outcomes into a common temporal framework that enables the determination of the net present value of the costs and outcome measures (Neumann et al., 2016). This ability to account for temporal patterns allows the decisionmaker to compare toxicity‐testing methodologies of different durations and explicitly incorporates the value of earlier test results.

CEA is a well‐established approach for designing therapeutic strategies (Lawrence, Robinson, & Miller, 2006; Neumann et al., 2016; Ryder, McDonough, Tosteson, & Lurie, 2009), improving medical diagnoses (Cebul & Posas, 1986), and is a cornerstone of health care budget resource allocation in health care systems (Russell et al., 1996). CEA has also been applied to the use of different toxicity‐testing methodologies. Omenn and Lave (1988) developed a CEA approach for evaluating the replacement of two‐year cancer bioassays with short‐term testing of mutagenicity endpoints. Gabbart and van Ierland (2010) investigated how CEA could be used to assist in tradeoffs between animal welfare, quality of information, and cost. They further discuss the value of generating a common metric that allows a quantitative tradeoff between these three elements. Norlen and colleagues promote the use of CEA in selection of a testing methodology focusing on tradeoffs between cost, duration, and uncertainty in the results (Norlen et al., 2014). Their article includes a case study comparing *in vivo* test methods for predicting acute systemic toxicity with a series of *in silico* and *in vitro* methods.

This article presents a framework for performing CEAs to select an optimum toxicity‐testing methodology for the large number of chemicals in commerce that currently have little or no toxicity data. The framework uses an approach similar to Norlen et al. (2014) but proposes a novel metric that captures the combined impacts of duration, cost, and uncertainty on the ability of regulators to make timely risk‐based decisions. The framework uses this metric as the outcome of the CEA. In this framework, a toxicity‐testing methodology is more cost effective when it enables a correct risk‐based decision at a lower cost and/or in less time. The framework also allows for the consideration of the characteristics of a specific decision when selecting a testing methodology. As a result, the framework can show that a methodology that is optimal for one type of decision may not be optimal for another type. The article illustrates the framework by creating two example CEAs. One for a simple risk‐based decision and a second for a complex decision.

As with all CEAs, the analyses created using this framework only consider the ability of a toxicity‐testing methodology to generate a correct decision and do not consider the costs and benefits that accrue from the ability to make correct decision. Such analyses of benefits and cost would be considered under value of information (VOI) analyses (Keisler, Collier, Chu, Sinatra, & Linkov, 2014). VOI analysis, however, require quantitative information that are not required by CEAs (e.g., costs of controls and the values of health benefits resulting from reductions in exposures). Thus, CEAs may be more readily applied to larger numbers of chemicals than VOI analyses.

## 2 FRAMEWORK FOR COST‐EFFECTIVENESS ANALYSES OF TOXICITY‐TESTING METHODOLOGIES

### 2.1 Introduction

The toxicity‐testing methodologies considered in the framework could consist of a single test, a battery of tests, or a tiered‐testing program. The costs considered in the evaluation framework include the costs of testing, but not the costs of analyzing the data or generating the specific toxicity findings used in regulatory decision making. The time considered is the time necessary to conduct the toxicity tests but not the time necessary to analyze the data or to issue regulations that reduce exposures.

CEA requires a clear definition of outcome. In this framework, outcome is linked to the benefits of toxicity testing. Benefits of testing occur because of the enablement of risk findings that provide a basis for assessing, and when appropriate, regulating chemical exposures. These benefits can be organized into four categories: (1) reduction of adverse effects from current exposures; (2) prevention of adverse effects from future exposures; (3) reduction of societal concerns by the conformation that current and future commercial uses of chemicals are safe; and (4) securing economic benefits from the use of chemicals incorrectly identified as being of concern. All these benefits require a determination of the acceptability of risks from different doses of the chemical. Therefore, within the context of this framework, the outcome is defined as the ability of risk managers to make “correct” risk‐based decisions for a tested chemical. As discussed below, risk‐based decisions are made using objective rules that link decisions to risk findings. A “correct” decision is defined as reaching the same decision under a given decision rule that would have been reached if the decisionmaker had perfect knowledge of the toxicity of the relevant chemical.

The outcome is further defined as the number of years in the time horizon where toxicity data are available to support correct decisions. The duration of this period is used as the outcome (rather than simply the date when the data first become available) because while some toxicity findings will be of immediate value (e.g., managing risks from existing exposures), others will find value in assessing exposures that occur from future uses of the chemicals. In addition, findings of safety for existing exposures provide an ongoing assurance to the exposed population that the chemicals in the products that they use have been evaluated and found to be safe. Thus, the benefits of toxicity data are best viewed as an ongoing resource that enables society to assess risks from current and future commercial use of chemicals.

The duration of toxicity testing affects the characterization of the net present costs and outcomes of testing. Since some toxicity tests may require multiple years to complete, a CEA will need to depreciate costs incurred in the future. Similarly, when the outcomes are delayed by toxicity‐testing methodologies with longer durations the value of the outcome will need to be discounted. To account for the effects of delay, a time horizon is defined for the CEA that is sufficiently long that it allows the measurement of both the costs and benefits of the toxicity‐testing methodology. In this article, future costs and outcomes are discounted using a fixed annual rate of reduction in value in the second and subsequent years of the time horizon.

### 2.2 Defining the Cost‐Effectiveness Ratio

The basis of a CEA is the cost‐effectiveness ratio (CER). Unlike many CEAs, the framework does not attempt to determine an incremental cost‐effectiveness ratio (ICER) for testing. An ICER measures changes in the CER that result from testing by comparing a revised CER to a CER based on the prior understanding of the toxicity of a chemical. In this framework, we assume that in the absence of toxicity testing no decision on the risk posed by an exposure to a chemical is possible and as a result no prior value of the CER can be determined. Once a chemical has been tested, however, the framework could be extended to include an ICER that evaluates the incremental benefits of additional testing. For example, if an *in silico* technique such as Wignall et al. (2018) is used to predict toxicity based on structure then an ICER could be performed to investigate the value of additional *in vitro* or *in vivo* testing.

In this framework, theCER for a single chemical is determined by dividing the cost of the testing by the outcome of the testing where both are adjusted for the times when the costs and outcomes occur. The preferred testing methodology is the methodology with the smallestCER. Let j=1,…,J denote the different testing methodologies. As discussed below, the CER is also a function of the regulatory decisions that use the toxicity data. Let l=1,…,L denote the different decisions. The value ofCER for the jth testing methodology and the lth decision ($\mathrm{CE}R^{j|l})\;$is then given by:

(1) $$\mathrm{CE}R^{j|l}=\frac{\sum_{y=1\;}^{y_{T,\;j}}\frac{C_{y}^{j}}{{\left(1+r\right)}^{y-1}}}{\sum_{y=y_{T,\;j\;}}^{y_{TH}}\frac{DMV_{y}^{j|l}}{{\left(1+r\right)}^{y-1}}},$$

where, $C_{y}^{j}\;$is the cost of performing the *j*th testing methodology in the yth year (millions of dollars); ${\mathrm{DMV}}_{y}^{j|l}$(Decision Making Value) is the probability of correctly making the *l*th type of regulatory decision in the yth year given the findings of the *j*th testing methodology (unitless); $y_{T,\;j}$ is the time it takes to perform the *j*th testing methodology (years); $y_{\mathrm{TH}}$ is the time horizon of the analysis, where $y_{\mathrm{TH}}$ must be greater than the largest $y_{T,j}$ of the methodologies evaluated (years); y is the time since the beginning of the toxicity testing (years); and  r is the annual discount rate (fraction reduced per year)

Uncertain toxicity data that increases the likelihood of incorrect decisions will generate higher values ofCER because DMV appears in the denominator ofCER and the values of DMV are reduced by uncertainty. In order to define DMV, the framework needs to link the toxicity data from a testing methodology to a decision‐making process. This is done by first linking the toxicity test data to a risk finding and then linking the risk finding to a decision‐making process. Once this relationship is in place, the uncertainty in the toxicity findings can be propagated through to the decision‐making process and the impact on decision making can be determined.

Table I presents an example derivation of CER for testing a chemical using a methodology taking ten years and costing five million dollars. The DMV for each year is 0.9 indicating that the estimate of toxicity has some uncertainty and could result in an incorrect decision 10% of the time. Assuming a discount rate of 3% and a time horizon of 20 years, the resulting value of CER is $0.85 million.

**Table I:** Example Derivation of the Cost‐Effectiveness Ratio (CER) for one Chemical and Toxicity‐Testing Methodology

Year	Event	Cost of Testing (Millions)	Discounted Cost of Testing (Millions)	Decision Making Value	Discounted Decision‐Making Value
1	Performing the test	$5.0	$5.0		
2			$0.0		
3			$0.0		
4			$0.0		
5			$0.0		
6			$0.0		
7			$0.0		
8			$0.0		
9			$0.0		
10			$0.0		
11	Using the data to assess risk		$0.0	0.9	0.670
12			$0.0	0.9	0.650
13			$0.0	0.9	0.631
14			$0.0	0.9	0.613
15			$0.0	0.9	0.595
16			$0.0	0.9	0.578
17			$0.0	0.9	0.561
18			$0.0	0.9	0.545
19			$0.0	0.9	0.529
20			$0.0	0.9	0.513
Net present value of cost and outcomes			$5.0		5.88
CER in millions of dollars per outcome			$0.85		

### 2.3 Characterizing Health Risks within the Framework

In this article, we illustrate the framework using a quantitative model of risk, R. The model assumes that the doses of a chemical necessary to cause a toxicological response and doses of the chemical received by the members of the exposed population both follow lognormal distributions (Chiu et al., 2018; Chiu & Slob, 2015). The distribution of doses occurs because of interindividual variation in exposures to one or more sources of the chemical. To calculate R, let x denote a dose of a chemical received over a specific duration of time and let f(x) be the probability density function of doses received by individuals in an exposed population over the duration. The cumulative distribution function, G(x), constitutes a population‐level dose–response function describing the incidence of adverse effects in the population if the entire population received a dose of *x* for the specific duration of time. With the assumption of a lognormal distribution for G(x), the dose–response function has a monotonically increasing and symmetric sigmoidal shape when viewed on the logarithmic scale, with the population incidence of 50% at the median (and geometric mean) of G(x). The function g(x), represents the probability density function associated with the interindividual variability in the bodyweight‐adjusted dose at, or above, which adverse effect(s) occur to the individual (often referred to as an individual's threshold dose). The function G(x) is hereafter referred to as a chemical's toxicity distribution. While the model assumes that individuals have thresholds, it does not assume that the population distributions have thresholds (e.g., G(x)is unbounded).

Let θ denote the set of parameters that determine the variability in the toxicity and exposure factors that determine the variability in the distribution of risk levels across individuals in an exposed population, where θ can be partitioned into separate parameters for the toxicity and exposure distribution, $\theta \;=[\theta_{\mathrm{tox}},\theta_{\exp}]\;$. The function f(x) is defined by a parametric distribution ($f\;\left(x|\theta_{\exp}\right)=\;LN\left(\mu_{\exp},\sigma_{\exp}\right)$) and the function g(x) is defined by a parametric distribution ($g\;\left(x|\theta_{\mathrm{tox}}\right)=\;LN\left(\mu_{\mathrm{tox}},\sigma_{\mathrm{tox}}\right))$, where the parameters $\mu_{\mathrm{tox}}$ and $\mu_{\exp}$ are the log_10_ values of the geometric means of g(x) and f(x) and $\sigma_{\mathrm{tox}}$ and $\sigma_{\exp}$ are the log_10_ values of the geometric standard deviations of g(x) and f(x). Assuming that individuals’ exposures and toxicity thresholds are statistically independent, the average risk for the population can be expressed as:

(2) $$\begin{array}{ccc} R & = & R\left(\theta \right)=E\left[G\left(x|\theta \right)\right]\\ & = & \int_{0}^{\infty }G\left(x|\theta_{\mathrm{tox}}\right)f\left(x|\theta_{\exp}\right)dx. \end{array}$$

**Fig 1:**
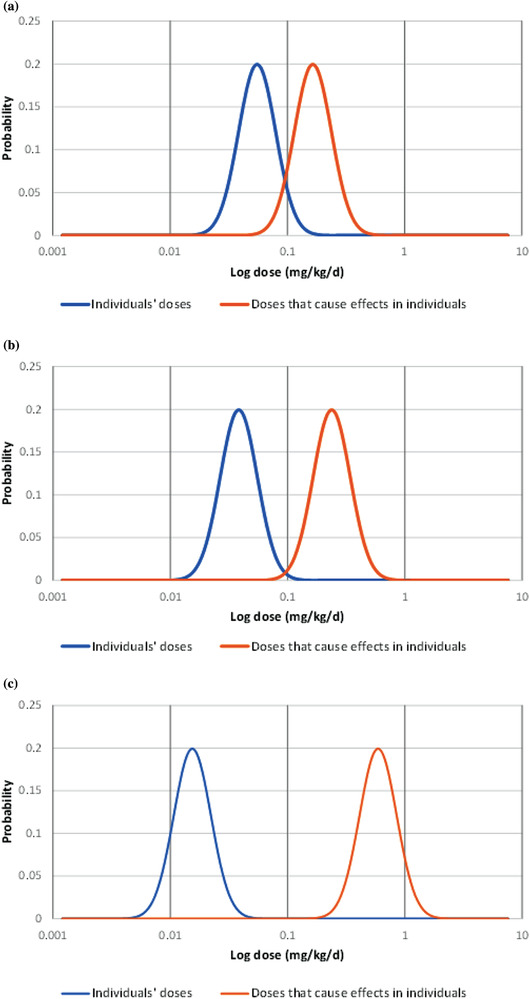
Risk model based on lognormal distributions (here expressed as probability density functions) of the variability in threshold of doses that cause effects in individuals in the population (toxicity distribution) and variability in doses received by individual members of the population (dose distribution). 1a. Lower bound of toxicity distribution overlaps with the upper bound of the dose distribution and risk is significant. 1b. Distributions slightly overlap, and risk is low. 1c. There is a gap between the upper bound of the exposure and lower bound of the toxicity distributions and risk is zero

Fig. 1 presents plots of g(x) and f(x) for three populations with different levels of risk. Larger risks occur when large fractions of the population receive doses that have a large probability of causing an adverse effect (the distributions overlap). The assumption of lognormality allows the determination of R for a population using the analytical solution to Equation (2).

(3) $$R=\Phi \left(\frac{\mu_{\exp}-\mu_{\mathrm{tox}}}{\sqrt{\sigma_{\exp}^{2}+\sigma_{\mathrm{tox}}^{2}}}\right),$$

where, Φ(·) denotes the cumulative distribution function of the standard normal distribution. The derivation of this solution is given in Appendix 1 of this article.

This approach differs from the hazard quotient and margin of exposure metrics commonly used in noncancer risk assessments (U.S. EPA, 1986) in that the value of R is the fraction of individuals in a population who experience one or more adverse effects resulting from exposures to a chemical that occur over a specific period of time. Examples of this approach have been developed using risk models based on quantitative aggregate exposure and adverse outcome pathways (Clewell et al., 2020; Hinderliter, Price, Bartels, Timchalk, & Poet, 2011; Hines, Edwards, Conolly, & Jarabek, 2018).

### 2.4 Characterizing Uncertainty in Toxicity, Exposure, and Risk

The lack of perfect information leads to uncertainty in estimates of $\theta_{\mathrm{tox}}$. Different testing methodologies will result in different estimates of $\theta_{\mathrm{tox}}$, each subject to different levels of measurement uncertainty. In this framework, the values of the parameters derived from a toxicity‐testing methodology are denoted as uncertain estimates $({\hat{\theta }}_{\mathrm{tox}}$) of the parameter values $\theta_{\mathrm{tox}}$. With *J* testing methodologies, there are *J* sets of estimates of the toxicity parameters ${\hat{\mu }}_{\mathrm{tox}}^{j}$ and ${\hat{\sigma }}_{\mathrm{tox}}^{j}$. If we assume that ${\hat{\mu }}_{\mathrm{tox}}^{j}\;$and ${\hat{\sigma }}_{\mathrm{tox}}^{j}$ have uncertainty distributions that are lognormal, the uncertainty from thejth methodology for toxicity testing can be characterized by the parameters $\mu \left({\hat{\mu }}_{\mathrm{tox}}^{j}\right),\sigma \left({\hat{\mu }}_{\mathrm{tox}}^{j}\right)$, $\mu ({\hat{\sigma }}_{\mathrm{tox}}^{j})$, and $\sigma ({\hat{\sigma }}_{\mathrm{tox}}^{j})$.

The relationships between ${\hat{\theta }}_{\mathrm{tox}}^{j}$ and $\theta_{\mathrm{tox}}$ for an individual chemical cannot be empirically determined by any toxicity test. Information on the uncertainty in the predictions of specific methodologies, however, can be characterized by the application of the methodology to chemicals where human toxicity data are available. Alternatively, information on the testing methodology itself can allow a characterization of the uncertainty in the values of ${\hat{\theta }}_{\mathrm{tox}}^{j}$. For example, error propagation should be possible in estimates of ${\hat{\theta }}_{\mathrm{tox}}^{j}$ based on the quantitative adverse outcome and aggregate exposure pathway models. As a result, the framework assumes that the analyst is able to develop an estimate of the relevant uncertainty parameters ($\mu \left({\hat{\mu }}_{\mathrm{tox}}^{j}\right),\sigma \left({\hat{\mu }}_{\mathrm{tox}}^{j}\right)$, $\mu ({\hat{\sigma }}_{\mathrm{tox}}^{j})$, and $\sigma ({\hat{\sigma }}_{\mathrm{tox}}^{j})$).

Regulatory actions reduce a population's exposure to a chemical by changing events in the exposure pathways for one or more sources of the chemical. These actions result in lower expected values of dose for the exposed population and values for exposure parameters that differ from the parameter values of the uncontrolled exposures. The parameters that describe the doses received from uncontrolled exposures in our framework are defined as $\mu_{0,\exp}$ and $\sigma_{0,\exp}$. The parameters that describe the doses that occur under the *K* regulatory actions are defined as $\mu_{k,\exp}$ and $\sigma_{k,\exp}$.

The exposure parameters under different regulatory actions as measured using a specific exposure assessment methodology will result in estimates of ${\hat{\mu }}_{0,\exp}$, ${\hat{\sigma }}_{0,\exp},$ ${\hat{\mu }}_{k,\exp}$, and ${\hat{\sigma }}_{k,exp}$ that are uncertain. These uncertainties would be characterized by the parameters $\mu ({\hat{\mu }}_{0,\exp})$, $\sigma ({\hat{\mu }}_{0,\exp})$, $\mu ({\hat{\sigma }}_{0,\exp})$, $\sigma ({\hat{\sigma }}_{0,\exp})$, $\mu ({\hat{\mu }}_{k,\exp})$, $\sigma ({\hat{\mu }}_{k,\exp})$, $\mu ({\hat{\sigma }}_{k,\exp})$, and $\sigma ({\hat{\sigma }}_{k,\exp})$. While the proposed framework can account for uncertainty in estimates of exposure and the CER can be determined for different exposure measurement methodologies, the focus of this article is on the application of CEA to the selection of a preferred toxicity‐testing methodology. The issue of selection of a methodology to characterize exposure (e.g., by modeling, analytical measurement, or other means) is, therefore, beyond the scope of the article. In this article, we make the simplifying assumption that there is a single method of determining the exposure parameters and the uncertainties in the estimates of the exposure parameters do not materially affect the uncertainty in the estimates of risk (${\hat{R}}_{0}^{j}$ and ${\hat{R}}_{k}^{j}$).

The estimated parameters derived from the jth toxicity‐testing methodology, ${\hat{\theta }}_{\mathrm{tox}}^{j}$, and the estimate of exposure under no action and the kth regulatory action, ${\hat{\theta }}_{0,\exp}$ and$\;{\hat{\theta }}_{k,\exp}$, are used in Equation (3) to generate estimates of values of risk for the exposed population (${\hat{R}}_{0}^{j}$ and ${\hat{R}}_{k}^{j}$) that reflect the choices of toxicity‐testing methodology, exposure measurement methodology, and regulatory action.

### 2.5 Use of Risk Findings in Regulatory Decision Making

The framework requires the definition of the decision‐making processes that use the risk findings and determines whether the findings based on ${\hat{\theta }}_{\mathrm{tox}}^{j}$ will result in the same decision as those based on $\theta_{\mathrm{tox}}$. Decisions reflect the characteristics of the sources of a chemical, the populations that are exposed, available controls, and the physicochemical, toxicokinetic, and toxicodynamic properties of the chemical. Regulatory decisions are also determined by the legal and regulatory frameworks for specific sources of exposure and specific populations (Krewski et al., 2014). Decisions can be made, for example, based on a target risk level (TRL) in an exposed population (U.S. EPA, 1991), a comparison of benchmarks of exposure to benchmarks of toxicity (U.S. EPA, 1991), or a balance between the costs of regulation and health benefits from reductions in exposures that result from regulation (Merkhofer, 2012).

**Fig 2:**
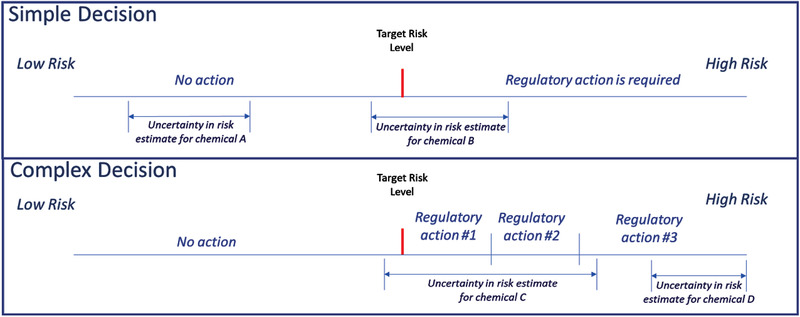
Simple and complex risk‐based decision making showing the choice set of actions and the impact of uncertainty in risk findings on decision making

In this article, the CEA framework is demonstrated using TRL‐based decisions. The simplest TRL‐based decision is the determination of whether a population with a given level of exposure has a risk that is above, or below, a TRL (Fig. 2). The decision is modeled using the following “simple decision rule” (SDR):

(4) $$\mathrm{SDR}\left(R|\mathrm{TRL}\right)=\{\begin{array}{c} 1\;\mathrm{if}\;R>\mathrm{TRL}\\ 0\;\mathrm{if}\;R\leq \mathrm{TRL} \end{array}.$$

Since R is unknown, the decision maker must make the decision using the estimate of uncontrolled risk that is based on the information provided by thejth toxicity‐testing methodology ${\hat{R}}_{0}^{j}$. This decision is expressed as:

(5) $$\mathrm{SDR}\left({\hat{R}}_{0}^{j}|\mathrm{TRL}\right)=\{\begin{array}{c} 1\;\mathrm{if}\;{\hat{R}}_{0}^{j}>\mathrm{TRL}\\ 0\;\mathrm{if}\;{\hat{R}}_{0}^{j}\leq \mathrm{TRL} \end{array}$$

Decisions become more complicated when the population risk exceeds the TRL and requires regulatory control. The following “complex decision rule” (CDR) is an example of these more complex decisions. Under this rule, the decisionmaker selects a regulatory action that reduces a population's risk to a level equal to, or below, the TRL. When there are multiple regulatory actions available that reduce the risk to levels equal to, or below, the TRL, the decisionmaker selects the action with the lowest cost. The rule also assumes that there will be at least one methodology that can reduce the risk to levels equal to, or below, the TRL. It should be also noted that only the rank of the costs of the regulatory actions is used in this process and not the specific costs. As with the SDR, the CDR is based on the estimated risks (in this case ${\hat{R}}_{0}^{j}$ and ${\hat{R}}_{k}^{j}$) and is expressed as:

(6) $$\mathrm{CDR}\;\left({\hat{R}}_{0}^{j}\mathrm{TRL}\right)=\left\{\begin{array}{c} k\;\;\mathrm{if}\;\left({\hat{R}}_{0}^{j}>\mathrm{TRL}\right)\cap \left({\hat{R}}_{k}^{j}\leq \mathrm{TRL}\right)\cap \left(k=\mathrm{argmin}\;AC_{k}\right)\\ 0\;\mathrm{if}\;{\hat{R}}_{0}^{j}\leq \mathrm{TRL} \end{array}\right.$$

where k is the selected regulatory action and $AC_{k}$ is the cost of the kth regulatory action.

This process of applying the CDR results in a selection of either no action or one of a series of increasingly stringent and increasingly costly regulatory actions that is a function of the size of $R_{0}$. These series of actions are termed by Norlen et al. (2014) as the “choice set” of actions. A choice set is created by the following process. Consider a population that is exposed to a chemical and has a value of $R_{0}$ that is two times the TRL. Assuming that there is a set of possible regulatory actions that reduce exposure and result in values of $R_{k}$ that are less than, or equal to, the TRL. Let regulatory action #1 be the action with the lowest cost and let regulatory action #1 reduce the population's exposures in such a way that it results in a fivefold reduction in population risk ( $R_{k\;=\;Action\;1}=R_{0}/5\;$). Under the CDR, regulatory action #1 would be selected for the population. Now consider a second population with a value of $R_{0}$ that is eight times the TRL. Regulatory action #1 would not be included in the pool of possible actions because $R_{k\;=\;\mathrm{Action}\;1}$ would exceed the TRL by a factor of 1.6. Let regulatory action #2 have the lowest cost of the smaller pool of actions with values of $R_{k}$ that are less than the TRL when $R_{0}$ is eight times the TRL. Because regulatory action #1 was the lowest cost action in the original pool and has now been excluded, the cost of the lowest cost action in the new pool (regulatory action #2) will be higher than regulatory action #1. Let regulatory action #2 reduce risk by a factor of 50. Now consider a third population where $R_{0}$ is 100 times the TRL. Regulatory action #2 would not be included in the pool of possible actions and a third action (regulatory action #3) would be selected. regulatory action #3 would be more be more effective, reducing risks by a factor of at least 100, and more expensive than regulatory action #2.

Regulatory action #1 is selected by the CDR for populations where $R_{0}$ is more than the TRL but less than five times the TRL; regulatory action #2 is selected where $R_{0}$ is more than five times the TRL but less than ten times the TRL; and regulatory action #3 is selected where $R_{0}$ is greater than ten times the TRL. Regulatory actions #1, #2, and #3 make up the choice set of K=3 actions for this chemical and its exposures. Risks of one, five, and ten times the TRL are values of risk where the selection of the regulatory action changes. These values are decision points (DPs) in the risk findings for the population.

### 2.6 Determining the $\mathrm{DM}V^{j|l}$ for the SDR and CDR

$\mathrm{DM}V^{j|l}$ is defined as the conditional probability of making a correct decision for the *l* ^th^ decision rule using information from the jth toxicity‐testing methodology, where the correct decision is the decision that would be made using $\theta_{\mathrm{tox}}$. $\mathrm{DM}V^{j|l}$for a simple decision rule ($\mathrm{DM}V^{j|\mathrm{SDR}}$) can be expressed as:

(7) $$\mathrm{DM}V^{j|\mathrm{SDR}}=P\left(\mathrm{DR}\left({\hat{R}}^{j}|\mathrm{TRL}\right)=\mathrm{DR}\left(R|\mathrm{TRL}\right)\right),$$

where $\mathrm{DR}({\hat{R}}^{j}|\mathrm{TRL})$ is the decision from a decision rule (DR) that is based on ${\hat{R}}^{j}$ and TRL. If the jth toxicity‐testing methodology perfectly predicts toxicity ( ${\hat{\theta }}_{\mathrm{tox}}^{j}=\;\theta_{\mathrm{tox}}$) the value of $\mathrm{DM}V^{j|\mathrm{SDR}\;}$is 1. The uncertainty in ${\hat{\theta }}_{\mathrm{tox}}^{j}$ translates into uncertainty in the estimates of ${\hat{R}}_{0}^{j}$ and ${\hat{R}}_{k}^{j}$ used in decision making and ultimately uncertainty in the choice of regulatory action. This uncertainty may reduce the probability of making a correct decision and if this occurs, the resulting values of $\mathrm{DM}V^{j|l}$ are less than 1.

The relationship between $\mathrm{DM}V^{j|l}$ and a given level of uncertainty in ${\hat{\theta }}_{\mathrm{tox}}^{j}$ varies with the nature of the decision. If the decision rule is the SDR (Equation 5), then $\mathrm{DM}V^{j|\mathrm{SDR}}$ is given by:

(8) $$\begin{array}{ccc} \mathrm{DM}V^{j|\mathrm{SDR}} & = & P\left[\left({\hat{R}}_{0}^{j}>\mathrm{TRL}\cap R_{0}>\mathrm{TRL}\right)\right.\\ & & \left.\cup \left({\hat{R}}_{0}^{j}\leq \mathrm{TRL}\cap R_{0}\leq \mathrm{TRL}\right)\right]. \end{array}$$

If the decision rule considers two risk findings, such as the CDR where there is a single regulatory action (*K* = 1), then there are three possible relationships between TRL and the DPs. First, TRL may be greater than $R_{0}$. Second, the TRL may be greater than $R_{K}$ but less than $R_{0}$. Finally, $R_{K}$ may be greater than TRL. As a result, $\mathrm{DM}V^{j|\mathrm{CDR}\;}$ is given by:

(9) $$\begin{array}{ccc} \;\mathrm{DM}V^{j|\mathrm{CDR}\;} & = & P\left[\left(\mathrm{TRL}\geq {\hat{R}}_{0}^{j}\;\cap \;\mathrm{TRL}\geq R_{0}\right)\;\right.\\ & & \left.\cup \;\left({\hat{R}}_{0}^{j}\geq \mathrm{TRL}\geq {\hat{R}}_{K}^{j}\;\cap \;R_{0}\geq \mathrm{TRL}\geq R_{K}\right)\;\right.\\ & & \left.\cup \;\left({\hat{R}}_{K}^{j}\geq \mathrm{TRL}\;\cap \;R_{K}\geq \mathrm{TRL}\right)\right], \end{array}$$

When there is more than one possible regulatory action in a choice set (K>1), $\mathrm{DM}V^{j|\mathrm{CDR}\;}$ is given by:

(10) $$\begin{array}{ccc} \;\mathrm{DM}V^{j|\mathrm{CDR}\;} & = & P\left[\left(\mathrm{TRL}\geq {\hat{R}}_{0}^{j}\;\cap \;\mathrm{TRL}\geq R_{0}\right)\cup \;\left({\hat{R}}_{0}^{j}\geq \mathrm{TRL}\geq {\hat{R}}_{1}^{j}\;\cap \;R_{0}\geq \mathrm{TRL}\geq R_{1}\right)\cup \cdots \cup \right.\\ & & \left.({\hat{R}}_{\left(K-1\right)}^{j}\geq \mathrm{TRL}\geq {\hat{R}}_{K}^{j}\;\cap \;R_{\left(K-1\right)}\;\geq \mathrm{TRL}\geq R_{K})\cup \;\left({\hat{R}}_{K}^{j}\geq \mathrm{TRL}\;\cap \;R_{K}\geq \mathrm{TRL}\right)\right], \end{array}$$

### 2.7 The Relationship of $\mathrm{CE}R^{j|l}$ and $\mathrm{DM}V^{j|l}$ with the Toxicity of the Chemical Being Tested

The value of $\mathrm{CE}R^{j|l}\;$varies with the toxicity of the chemical being tested. As a result, a toxicity‐testing methodology that has the lowest $\mathrm{CE}R^{j|l}$ for one chemical may not have the lowest $\mathrm{CE}R^{j|l}$ for a second chemical. The relationship between $\mathrm{CE}R^{j|l}$ and toxicity is determined by the relationship between $\mathrm{DM}V^{j|l}$ and $R_{0}$ and $R_{k}$ and by extension with θ.

An example of how $\mathrm{DM}V^{j|l}$ varies with θ is given in Fig. 2. The figure presents plots of the simple and complex decisions for four chemicals (A–D) each with different θ. In the figure, the population risk is presented on the *x* axis and the decision points and TRL are identified by vertical lines. The range of uncertainty in ${\hat{R}}_{0}^{j}$ resulting from the uncertainty in ${\hat{\theta }}_{\mathrm{tox}}^{j}$ is presented for each of the four chemicals. In this figure, chemicals A and B have similar degrees of uncertainty in the estimates of ${\hat{R}}_{0}^{j},$ but have different values of $\mathrm{DM}V^{j|\mathrm{SDR}}$. The range of ${\hat{R}}_{0}^{j}$values for chemical A are all below the TRL. Since the corresponding uncertainty in ${\hat{R}}_{0}^{j}$ does not affect the finding that the risk is below the TRL, the uncertainty in ${\hat{\theta }}_{\mathrm{tox}}^{j}$ for chemical A has no impact on the decision and the value of $\mathrm{DM}V^{j|\mathrm{SDR}\;}$ is 1. In contrast, chemical B's range of ${\hat{R}}_{0}^{j}$ values include levels that are above and below the TRL. As a result, some of the estimates of ${\hat{R}}_{0}^{j}$ for chemical B led to an incorrect decision. (Since $R_{0}$ is a constant, it cannot be both above and below the TRL.) This results in a value of $\mathrm{DM}V^{j|\mathrm{SDR}}$that is less than 1. As decisions become more complex, the impact of uncertainty in ${\hat{\theta }}_{\mathrm{tox}}^{j}$ on $DMV^{j|l\;}$ increases. In the complex decision shown by Fig. 2, the decisionmaker needs to assign the population's risk into one of four categories that define whether the exposure needs to be controlled and if so, the level of control required. When the values of ${\hat{\theta }}_{\mathrm{tox}}^{j}$ are uncertain and when small differences in toxicity parameter values affect a decision, the probability of a correct assignment can be low. For chemical C, the uncertainty in ${\hat{\theta }}_{\mathrm{tox}}^{j}$ is sufficiently large to make it unclear which of the four possible levels of control are required. As a result, there will be a low value of $\mathrm{DM}V^{j|\mathrm{CDR}\;}$for chemical C. Finally, it is also possible for uncertainty in ${\hat{\theta }}_{\mathrm{tox}}^{j}$to become less important for highly toxic chemicals. The estimate of toxicity for chemical D in Fig. 2, while uncertain, clearly requires the most stringent level of control (regulatory action #3) and would have a $\mathrm{DM}V^{j|\mathrm{CDR}\;}$ of 1.

The finding that the cost‐effectiveness ratio of a toxicity‐testing methodology applied to a given risk‐based decision will vary across chemicals with different toxicities and/or different exposures suggests that the selection of a toxicity‐testing methodology will not result in the identification of a single preferred methodology for all untested chemicals. As a result, a testing program may find it beneficial to investigate which methodology is preferred for a chemical. This point is examined in more detail in the discussion section below.

## 3 ILLUSTRATIVE APPLICATIONS OF THE FRAMEWORK

To demonstrate how the proposed framework operates, we present two example CEAs that address the use of toxicity data in the SDR and CDR described above. Data from five hypothetical toxicity‐testing methodologies (j=1,…,5) are used, resulting in five values of CER for each of the two decision rules $\mathrm{CE}R^{j\;=\;1,\text{…},5|\mathrm{SDR}}$ and $\mathrm{CE}R^{j\;=\;1,\text{…},5|\mathrm{CDR}}$.

The purpose of the applications is not to select the best of the hypothetical methodologies (although the results could be used for this purpose), nor are any of the five methodologies intended to represent an existing or new testing methodology. Instead, the characteristics of the five methodologies are designed to explore the impacts of differences in the cost of the tests, the duration of the tests, and the level of uncertainty in the values of ${\hat{\theta }}_{\mathrm{tox}}^{j}$ that are derived from the test results. The goal of the analysis is to demonstrate how the framework assesses cost, duration and uncertainty and the impact of specific changes in these factors on the values of CER for groups of chemicals with a wide range of toxicity.

In these examples, we assume that a regulatory agency is tasked with assessing the toxicity of a large number of chemicals that have varying levels of toxicity. While Equation (1) provides estimates of the cost‐effectiveness of a testing strategy for an individual chemical, an actual program of testing chemicals in commerce would test hundreds or thousands of chemicals. Therefore, in these examples we evaluate the cost‐effectiveness of a testing approach for large numbers of chemicals that have varying levels of toxicity. We make the simplifying assumption that the same methodology is used for each chemical and the cost of testing will be similar across the chemicals tested. The values of CER generated in these examples are the cost of the program divided by the sum of the outcomes for the tested chemicals over the time horizon.

### 3.1 Design of the Illustrative Examples

The toxicity testing program in the illustrative examples is assumed to have the following characteristics. The program is funded on an annual basis at a constant amount over the period spanned by the time horizon and the cost of testing a chemical remains constant for each of the methodologies for the same period. The data become available when the testing of the chemical using the *j*th toxicity‐testing methodology, $y_{T,\;j}$ is completed. TheDMV and the cost of testing are reduced by the same annual discount rate for year 2 and later years. The program starts testing a new group of chemicals each year. The cost of the toxicity testing is fully funded using the budget that is available the year the test is initiated. The size of the group is a function of the cost of the testing of a single chemical and the annual budget.

As described above, for a fixed initial exposure, chemicals with higher toxicity will pose larger values of $R_{0}$ and $R_{k}$. To maintain values of $R_{k}$ that are no more than the TRL requires more stringent controls for chemicals with higher toxicity. Therefore, the values of $\mathrm{CE}R^{j|\mathrm{CDR}}$ vary across chemicals with different toxicities and a range of toxicity values are used to explore how this variation occurs. In the CDR we assume that there are three regulatory actions in the choice set (K=3). The values of ${\hat{\theta }}_{0,\;\exp}$and ${\hat{\theta }}_{k,\;\exp}$ are defined for the uncontrolled exposures and for the exposures under each of the three regulatory actions, respectively. For simplicity, the uncertainties in ${\hat{\theta }}_{0,\;\exp}$ and ${\hat{\theta }}_{k,\;\exp}$ are not considered in these analyses. The values of ${\hat{\theta }}_{\mathrm{tox}}^{j}$, ${\hat{\theta }}_{0,\;\exp},\;$and ${\hat{\theta }}_{k,\;\exp}$ are used to determine values of ${\hat{R}}_{0}^{j}$ and ${\hat{R}}_{k}^{j}$. These values along with information on the costs and durations of the testing are used to determine the values of $\mathrm{CE}R^{j\;=\;1,\text{…},5|\mathrm{SDR}}$ and $\mathrm{CE}R^{j\;=\;1,\text{…}5|\mathrm{CDR}}$.

The values of the parameters that are common to the two CEAs are given in Table II. In these examples we assume that the critical effect for the chemicals is severe and the value of TRL is therefore set at 10^–6^. Different values for TRL could be used depending on the severity of a chemical's endpoint. Table III provides the values of parameters for the five toxicity‐testing methodologies. The values for the exposure parameters for the choice set of regulatory actions are shown in Table IV. Regulatory action #1 reduces the true population risk by a factor of five, regulatory action #2 by a factor of 10, and regulatory action #3 reduces the true risk for all chemicals to a level below the TRL.

**Table II:** Parameters Used in the Two Example CEAs

Parameter	Description	Value	Unit
TH	Time horizon	11–20	Years
	Total annual budget for toxicity testing	10	Millions $/year
r	Annual discount rate	3	Percent
TRL	Target risk level	$10^{-6}$	(Unitless)
$\mu_{\mathrm{tox}}$	Range of values of log_10_ of geometric mean of toxicity distribution	−5–2	Log_10_ (mg/kg/day)
$\mu ({\hat{\mu }}_{\mathrm{tox}}^{j}-\mu_{\mathrm{tox}})$	Bias in the estimate of $\mu_{\mathrm{tox}}$	0	(Unitless)
${\hat{\sigma }}_{\mathrm{tox}}$	Log_10_ geometric standard deviation of toxicity distribution	1	(Unitless)

**Table III:** Parameter Values for the Duration, Cost, and Uncertainty for the Five Toxicity‐Testing Methodologies

			Toxicity‐Testing Methodology				
			1	2	3	4	5
Parameter	Description	Units	Base Case	Reduced Cost	Reduced Time	Reduced Uncertainty	All Reduced
$y_{T,\;j}$	Duration of toxicity testing	Years	10	10	2	10	2
$C^{j}$	The total cost of toxicity testing one chemical	Millions $	5	1	5	5	1
$\sigma ({\hat{\mu }}_{\mathrm{tox}}^{j})$	Uncertainty in the geometric standard deviation about the mean of toxicity after testing	Unitless	1	1	1	0.2	0.2

**Table IV:** Exposure Parameter Values for the Choice Set of Regulatory Actions

				Regulatory Actions		
Parameter	Description	Units	No action	1	2	3
${\hat{\mu }}_{k,\;\exp}$	Log_10_ of geometric mean of exposure distribution	Log_10_ (mg/kg/day)	−8	−8.5	−8.8	−14
${\hat{\sigma }}_{k,\;\exp}$	Log_10_ of geometric standard deviation of exposure distribution	Log_10_ (mg/kg/day)	0.5	0.4	0.4	0.1

The parameter values for the toxicity‐testing methodologies are designed to explore the impacts of changes in cost, testing duration, and uncertainty (Table II). Toxicity‐testing methodology #1 provides a base case for comparison to the remaining four methodologies. Toxicity‐testing methodology #2 differs from the base case by having a fivefold reduction in cost, toxicity‐testing methodology #3 differs by a fivefold reduction in the duration of testing, and toxicity‐testing methodology #4 differs by a fivefold reduction in the uncertainty in the estimates of toxicity. The reduction in the uncertainty is modeled by reducing the value of $\sigma (\mu_{\mathrm{tox}})$ by a factor of five. Toxicity‐testing methodology #5 differs from the base case by a combination of a fivefold reduction in cost, duration, and $\sigma (\mu_{\mathrm{tox}})$.

As discussed in Appendix B, the selection of the time horizon (TH) influences the relationship between duration of testing and the value of the CER. The shortest TH possible in the two examples is 11 years. This is the minimum length of time needed for generating toxicity data for toxicity‐testing methodologies #1, #2, and #4 and for promulgating regulations that use the data. The maximum value of TH used in the examples is 20 years. This upper limit for the value of TH is selected because it is expected that the methodologies used by testing programs will evolve over time, making the assumption that current toxicity‐testing methodologies will be used without modification for more than 20 years unlikely.

### 3.2 Impact of Variability and Uncertainty in $\mu_{\mathrm{tox}}$ on DMV and CER

**Fig 3:**
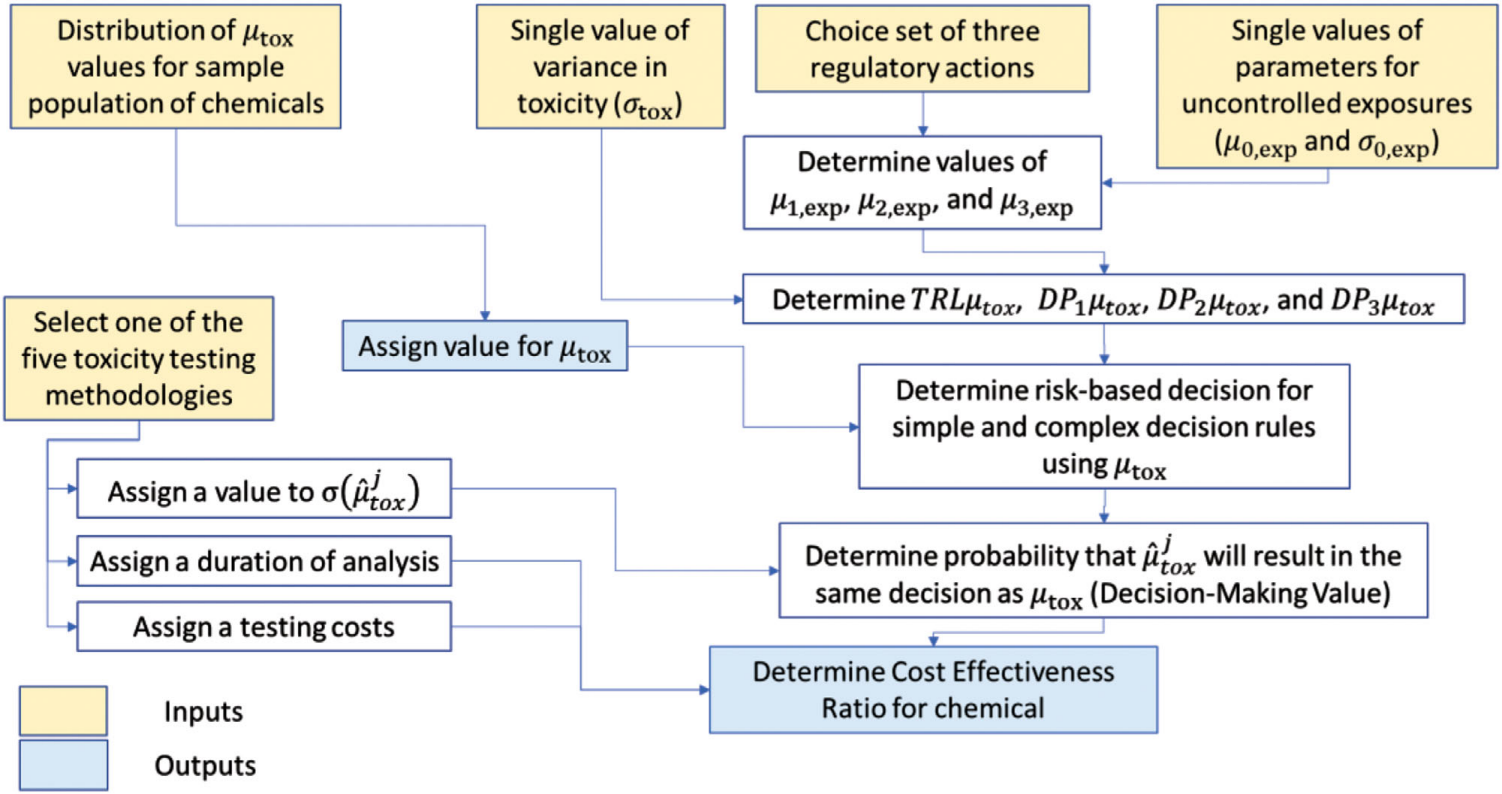
Flowchart for the modeling of CER for a single chemical. In the illustrative examples this is repeated for 5,000 chemicals. Inputs to the process are taken from Tables II–IV

Fig. 3 presents the process of determining the values for $\mathrm{DM}V^{j|l}$ and $\mathrm{CE}R^{j|l}$ for a chemical. The determination of the impact of uncertainty on $\mathrm{DM}V^{j|\mathrm{SDR}}$ and $\mathrm{DM}V^{j|\mathrm{CDR}}$ is made by first assigning a true value to $\mu_{\mathrm{tox}}$ and then adding uncertainty from the methodology‐specific measurement error to produce a value of ${\hat{\mu }}_{\mathrm{tox}}^{j}$. The probability of $\mu_{\mathrm{tox}}$ and ${\hat{\mu }}_{\mathrm{tox}}^{j}$ giving the same decisions (i.e., $\mathrm{DM}V^{j|\mathrm{SDR}}$ and $\mathrm{DM}V^{j|\mathrm{CDR}}$) is then determined based on the uncertainty in the measurement and the nature of the decision. This process is repeated using a wide range of values for $\mu_{\mathrm{tox}}$. All modeling is performed using Excel software.

In this article, values of $\mu_{\mathrm{tox}}$ for the tested chemicals are assumed to follow a log uniform distribution bounded by −5 and 2 (0.00001 to 100 mg/kg/day). We note that this range is likely to be wider than the expected range of chemical toxicities, but the wide range is helpful in demonstrating the relationship between CER and $\mu_{\mathrm{tox}}$. A value of $\mu_{\mathrm{tox}}$ is randomly selected from this range and values of $\mathrm{DM}V^{j|\mathrm{SDR}}$ and $\mathrm{DM}V^{j|\mathrm{CDR}}$are determined using the processes described below. The process is repeated 5,000 times to produce a data set of matched values of $\mu_{\mathrm{tox}}$, $\mathrm{DM}V^{j|\mathrm{SDR}},$ $\mathrm{DM}V^{j|\mathrm{CDR}}$, $\mathrm{CE}R^{j|\mathrm{SDR}},$ and $\mathrm{CE}R^{j|\mathrm{CDR}}.$

The first step in the process is to determine the value of $\mu_{\mathrm{tox}}$ that corresponds to the TRL ($\mathrm{TRL}\mu_{\mathrm{tox}}$). This value can be derived using Equation (3) and solving for $\mathrm{TRL}\mu_{\mathrm{tox}}$:

(11) $$\mathrm{TRL}\mu_{\mathrm{tox}}\;=\;\mu_{\exp}\;-\;\left(\Phi^{-1}\left(\mathrm{TRL}\right)\times \sqrt{\sigma_{\exp}^{2}+\sigma_{\mathrm{tox}}^{2}}\right).$$

As stated above, in these illustrative examples we assume that the uncertainty in $\mu_{\exp}$,$\;\sigma_{\exp}$, and $\sigma_{\mathrm{tox}}$ are small and can be treated as being zero. Thus, there is a single value of $\mathrm{TRL}\mu_{\mathrm{tox}}$ for any given value of TRL. Since the value of $\mu_{\mathrm{tox}}$ has been defined, its relationship to $\mathrm{TRL}\mu_{\mathrm{tox}}$is known. When $\mu_{\mathrm{tox}}>\mathrm{TRL}\mu_{\mathrm{tox}}$, then:

(12) $$\;\mathrm{DM}V^{j|\mathrm{SDR}}=\;P\left({\hat{\mu }}_{\mathrm{tox}}^{j}>\mathrm{TRL}\mu_{\mathrm{tox}}\;\right)$$

The probability that ${\hat{\mu }}_{\mathrm{tox}}^{j}>\mathrm{TRL}\mu_{\mathrm{tox}}$ can be determined based on the assumption of lognormality for the uncertainty distribution for ${\hat{\mu }}_{\mathrm{tox}}^{j}$. Let Φ(x,μ,σ) denote the cumulative distribution function of a normal distribution with a mean of μ and standard deviation of σ for a value x. Let μ equal ${\hat{\mu }}_{\mathrm{tox}}^{j}$, σ equal $\sigma ({\hat{\mu }}_{\mathrm{tox}}^{j})$, and *x* equal $\mathrm{TRL}\mu_{\mathrm{tox}}$. $\mathrm{DM}V^{j|\mathrm{SDR}}$ is then given by:

(13) $$P\;\left({\hat{\mu }}_{\mathrm{tox}}^{j}>\mathrm{TRL}\mu_{\mathrm{tox}}\;\right)=\;\Phi \left(\mathrm{TRL}\mu_{\mathrm{tox}},{\hat{\mu }}_{\mathrm{tox}}^{j},\sigma \left({\hat{\mu }}_{\mathrm{tox}}^{j}\right)\right).$$

Similarly, when $\mu_{\mathrm{tox}}\leq \mathrm{TRL}\mu_{\mathrm{tox}},$ we have:

(14) $$P\;\left({\hat{\mu }}_{\mathrm{tox}}^{j}\leq \mathrm{TRL}\mu_{\mathrm{tox}}\;\right)=\;1-\;\Phi \left(\mathrm{TRL}\mu_{\mathrm{tox}},\;{\hat{\mu }}_{\mathrm{tox}}^{j},\sigma \left({\hat{\mu }}_{\mathrm{tox}}^{j}\right)\right).$$

The complete equation for $\mathrm{DM}V^{j|\mathrm{SDR}}$ is then:

(15) $$\begin{array}{c} \mathrm{DM}V^{j|\mathrm{SDR}}=I_{\left[\mu_{\mathrm{tox}}>\mathrm{TRL}\mu_{\mathrm{tox}}\right]}\Phi \left(\mathrm{TRL}\mu_{\mathrm{tox}},{\hat{\mu }}_{\mathrm{tox}}^{j},\sigma \left({\hat{\mu }}_{\mathrm{tox}}^{j}\right)\right)+\;I_{\left[\mu_{\mathrm{tox}}\leq \mathrm{TRL}\mu_{\mathrm{tox}}\right]}\left(1-\Phi \left(\mathrm{TRL}\mu_{\mathrm{tox}},\;{\hat{\mu }}_{\mathrm{tox}}^{j},\sigma \left({\hat{\mu }}_{\mathrm{tox}}^{j}\right)\right)\right), \end{array}$$

where $I_{\left[\cdot \right]}$ denotes an indicator function for the condition [·]. Using Equation (15), the value $\mathrm{DM}V^{j|\mathrm{SDR}}$ is determined for each value of $\mu_{\mathrm{tox}}$.

The relationship between the values of $\mathrm{DM}V^{j|\mathrm{CDR}}$ and $\mu_{\mathrm{tox}}$ is a function of both the TRL and the decision points in the risk findings. As discussed above, the decision point is defined as the largest value of $R_{0}$ that can be reduced by an action to a level at, or below, the TRL and is the risk where the choice of action changes. Let $DP_{1}$be the decision point for regulatory action 1 and let $DP_{1}\mu_{\mathrm{tox}}$ be the value of $\mu_{\mathrm{tox}}$ that corresponds to $DP_{1}$. The value of $DP_{1}\mu_{\mathrm{tox}}$ is given by:

(16) $$DP_{1}\mu_{\mathrm{tox}}\;=\;\mu_{\exp}-\left(\Phi^{-1}\left(DP_{1}\right)\times \sqrt{\sigma_{\exp}^{2}+\sigma_{\mathrm{tox}}^{2}}\right).$$

The corresponding equation for $\mathrm{DM}V^{j|\mathrm{CDR}}$ where (*K* = 3) is then:

(17) $$\begin{array}{ccc} \mathrm{DM}V^{j|\mathrm{CDR}} & = & I_{\left[\mu_{\mathrm{tox}}\leq \mathrm{TRL}\mu_{\mathrm{tox}}\right]}\left[1-\Phi \left(\mathrm{TRL}\mu_{\mathrm{tox}},{\hat{\mu }}_{\mathrm{tox}}^{j},\sigma \left({\hat{\mu }}_{\mathrm{tox}}^{j}\right)\right)\right]\\ \; & & +\;I_{\left[\mathrm{TRL}\mu_{\mathrm{tox}}\leq \mu_{\mathrm{tox}}\leq \;DP_{1}\mu_{\mathrm{tox}}\right]}\left(\Phi \left(DP_{1}\mu_{\mathrm{tox}},{\hat{\mu }}_{\mathrm{tox}}^{j},\sigma \left({\hat{\mu }}_{\mathrm{tox}}^{j}\right)\right)-\Phi \left(\mathrm{TRL}\mu_{\mathrm{tox}},\;{\hat{\mu }}_{\mathrm{tox}}^{j},\sigma \left({\hat{\mu }}_{\mathrm{tox}}^{j}\right)\right)\right)\\ & & +\;I_{\left[DP_{1}\mu_{\mathrm{tox}}\leq \mu_{\mathrm{tox}}\leq \;DP_{2}\mu_{\mathrm{tox}}\right]}\left(\Phi \left(DP_{2}\mu_{\mathrm{tox}},{\hat{\mu }}_{\mathrm{tox}}^{j},\sigma \left({\hat{\mu }}_{\mathrm{tox}}^{j}\right)\right)-\Phi \left(DP_{1}\mu_{\mathrm{tox}},{\hat{\mu }}_{\mathrm{tox}}^{j},\sigma \left({\hat{\mu }}_{\mathrm{tox}}^{j}\right)\right)\right)\\ & & +\;I_{\left[DP_{2}\mu_{tox}\leq \mu_{tox}\leq \;DP_{3}\mu_{tox}\right]}\left(\Phi \left(DP_{3}\mu_{\mathrm{tox}},{\hat{\mu }}_{\mathrm{tox}}^{j},\sigma \left({\hat{\mu }}_{\mathrm{tox}}^{j}\right)\right)-\Phi \left(DP_{2}\mu_{\mathrm{tox}},{\hat{\mu }}_{\mathrm{tox}}^{j},\sigma \left({\hat{\mu }}_{\mathrm{tox}}^{j}\right)\right)\right)\\ & & +\;I_{DP_{3}\mu_{tox}\leq \mu_{tox}}\Phi \left(DP_{3}\mu_{\mathrm{tox}},{\hat{\mu }}_{\mathrm{tox}}^{j},\sigma \left({\hat{\mu }}_{\mathrm{tox}}^{j}\right)\right). \end{array}$$

The values of $\mathrm{DM}V^{j|\mathrm{SDR}}$ and $\mathrm{DM}V^{j|\mathrm{CDR}}$ are determined for a chemical using Equations (15) and (17), the methodology‐specific values of $\sigma ({\hat{\mu }}_{\mathrm{tox}}^{j})$, and values in Tables II, III, and IV.

Once the value of $\mathrm{DM}V^{j|\mathrm{SDR}}$ and $\mathrm{DM}V^{j|\mathrm{CDR}}$ are determined, the corresponding values of $\mathrm{CE}R^{j|\mathrm{SDR}}$ and $\mathrm{CE}R^{j|\mathrm{CDR}}$are calculated using Equation (1). The impact of the different durations and costs of the testing methodologies are modeled using a table (similar to Table I) that determines the net present values of costs and outcomes over the time horizon of the analysis.

## 4 RESULTS

**Fig 4:**
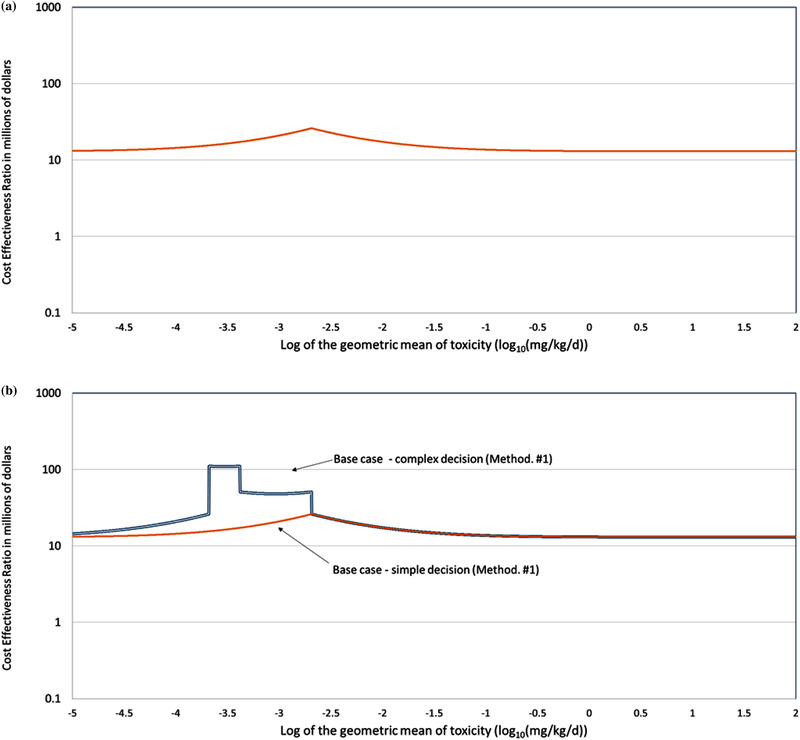
Changes in the cost‐effectiveness ratio (CER) of toxicity‐testing methodology #1 (base case) across 5,000 simulated chemicals of different toxicological potencies ($\mu_{\mathrm{tox}}$) for the simple decision rule (4a) and for both the simple and complex decision rule (4b)

**Fig 5:**
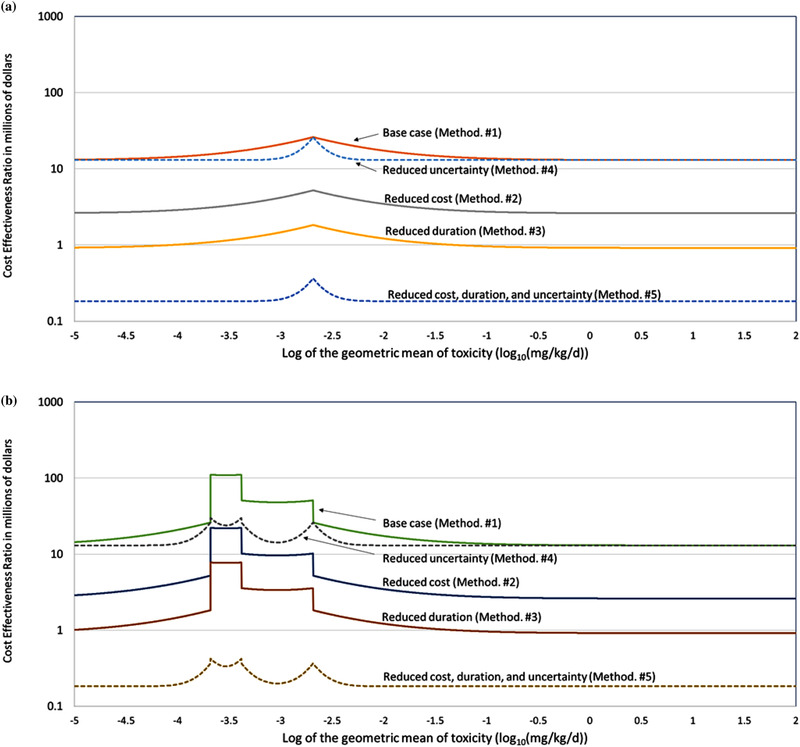
Comparison of changes in the cost‐effectiveness ratio (CER) of the five toxicity‐testing methodologies across 5,000 simulated chemicals of different toxicological potencies ($\mu_{\mathrm{tox}}$) for a simple decision rule (5a) and a complex decision rule (5b)

Figs. 4 and 5 give the estimates of CER for 5,000 chemicals of varying toxicity. Fig. 4a presents the change in CER for the SDR using data from toxicity‐testing methodology #1 (base case). The changes in the value of CER with $\mu_{\mathrm{tox}}$ occur as a result of the decreases of $\mathrm{DM}V^{j|\mathrm{SDR}},$ and $\mathrm{DM}V^{j|\mathrm{CDR}}$ for chemicals with values of $\mu_{\mathrm{tox}}$ that are close to $\mathrm{TRL}\mu_{\mathrm{tox}}$ (because the DMV occurs in the denominator of the CER [Equation (1)], these decreases results in increases in the values of CER). The decreases in DMV occur at such values of $\mu_{\mathrm{tox}}$ because at such values even small differences between $\mu_{\mathrm{tox}}$ and ${\hat{\mu }}_{\mathrm{tox}}^{j}$ can result in an incorrect decision.

Fig. 4b shows the difference between the simple and complex decisions for toxicity‐testing methodology #1 (base case). The two decisions have the same values of CER for chemicals with values of $\mu_{\mathrm{tox}}$ that are greater than $\mathrm{TRL}\mu_{\mathrm{tox}}$. For values that are less than the $\mathrm{TRL}\mu_{\mathrm{tox}},$ the values of $\mathrm{CE}R^{j|\mathrm{CDR}}$ diverge from $\mathrm{CE}R^{j|\mathrm{SDR}}$ with the values of $\mathrm{CE}R^{j|\mathrm{CDR}}$ increasing steeply. This occurs because a given level of uncertainty in values of the toxicity parameters leads to incorrect decisions more often with the CDR than with the SDR for such chemicals.

Figs. 5a and 5b present the variation in CER with toxicity for the SDR and CDR. Toxicity‐testing methodology #1 (base case) has the largest number of undesirable characteristics (higher cost, longer testing duration, and higher uncertainty) of the five methodologies considered. As a result, the values of $\mathrm{CE}R^{1}$ are the highest of the five methodologies for both the SDR and CDR. Toxicity‐testing methodology #5 has the largest number of desirable characteristics and the values of $\mathrm{CE}R^{5}$ are the lowest of the methodologies for both SDR and CDR.

In Fig. 5b, the variation of $\mathrm{CE}R^{j|\mathrm{CDR}}$ with the toxicity of the chemical appears as a series of separate peaks when the toxicity‐testing methodology has low uncertainty (toxicity‐testing methodologies #4 and #5) and as a step function when the methodology has higher uncertainty (toxicity‐testing methodologies #1, #2, and #3). Both the peaks and the edges of the steps occur at the values of $\mu_{\mathrm{tox}}$ that correspond to the TRL and the decision points (i.e., $\mathrm{TRL}\mu_{\mathrm{tox}}$ and $DP_{k}\mu_{\mathrm{tox}}$). At higher uncertainties, the shape is driven by the relative difficulty of identifying the correct regulatory action. The ability of a testing methodology to generate a toxicity estimate that identifies the correct action is smaller when the range of $\mu_{\mathrm{tox}}$ values that require a certain regulatory action is small. This can be seen in Fig. 4b where the largest values of CER occur for values of $\mu_{\mathrm{tox}}$ where regulatory action #2 is the correct action. Regulatory action #2 is only correct for a small range of values of $\mu_{\mathrm{tox}}$ (0.0002–0.0004 mg/kg/day). Regulatory action #1 is correct for a larger range (0.0004–0.002 mg/kg/day) and the values of CER for that action are lower.

In these examples, we assume a fixed budget for testing. Reducing the cost of testing for a single chemical (toxicity‐testing methodology #2), therefore, increases the number of chemicals tested and increases the output (number of chemicals and years that the results are available) by a factor of five. As a result, the fivefold decrease in cost reduces the value of CER by a factor of five. This decrease is independent of the values of the toxicity parameters of the chemicals and the decision‐making rule.

The reduction in testing duration by a factor of five (toxicity‐testing methodology #3) also resulted in a reduction in the value of CER. Like the impact of a reduction in cost, this decrease is independent of the values of the toxicity parameters of the chemicals and the decision‐making rule. Unlike cost, the impact of the reduced duration of testing is not direct but is mediated through its impact on the times when the test findings are available.

As discussed in Appendix B, the size of the impact is affected by $y_{T,\;1}$, r, and TH, with the value of TH having the largest effect. There is no “correct” value for TH. In this assessment we use a range of values to determine the sensitivity of the results to the assigned value of TH. As shown in Appendix B, at the lowest value of TH (11 years), the value of CER decreases 49‐fold from a fivefold reduction in duration. At the largest value of TH (20 years), the reduction is 3.4‐fold. Since it is not clear which value of TH is most appropriate, the plots of CER in Figs. 4 and 5 use the average value of CER for values of TH of 11 through 20 years (a 14‐fold reduction).

Reducing the uncertainty in the toxicity data ($\sigma ({\hat{\mu }}_{\mathrm{tox}}^{j})$) fivefold (toxicity‐testing methodology #4) lowers the value of the CER by raising the values of DMV that had been affected by the uncertainty in ${\hat{\mu }}_{\mathrm{tox}}^{j}$. As discussed in Section 2.2, the value of DMV varies with the chemical‐specific value of $\mu_{\mathrm{tox}}$ and changes in DMV are limited to chemicals with values of $\mu_{\mathrm{tox}}$ that are close to the $\mathrm{TRL}\mu_{\mathrm{tox}}$. This can be seen in Figs. 4b and 5b where the values of CER for chemicals with low toxicity ($\mu_{\mathrm{tox}}$>$\mathrm{TRL}\mu_{\mathrm{tox}}$) are similar for both levels of uncertainty.

The average and maximum values of $\mathrm{CE}R^{j,l}$ for the range of chemical toxicities for each of the methodologies and decisions are given in Tables V and VI. The average values are an appropriate measure for selecting toxicity‐testing methodologies for programs testing large numbers of chemicals and where the chemicals have a wide range of toxicity. The maximum value of $\mathrm{CE}R^{j,l}$ provides an upper bound for the chemicals most affected by the reduction in uncertainty. The fivefold reduction in $\sigma (\mu_{\mathrm{tox}})$ results in a 1.1‐fold reduction in the average value of $\mathrm{CE}R^{j,l}$ for the 5,000 chemicals under the SDR and 1.6‐fold reduction under the CDR. The largest reduction in any of the 5,000 chemicals is 1.5 and 4.6‐fold for the SDR and CDR, respectively.

**Table V:** Average and Maximum Values of the Cost‐Effectiveness Ratio (CER) for Each Toxicity‐Testing methodology Across the Range of Toxicological Potencies ($\mu_{\mathrm{tox}}$). Lower Values Reflect Greater Cost Effectiveness

Toxicity‐Testing Methodology	Average CER Across all Values of $\mu_{\mathrm{tox}}$		CER for the Value of $\mu_{\mathrm{tox}}$ most Impacted by Uncertainty	
	Simple Decision	Complex Decision	Simple Decision	Complex Decision
Toxicity‐testing methodology #1 (Base case)	15	23	26	110
Toxicity‐testing methodology #2 (Less cost)	3.1	4.5	5.2	22
Toxicity‐testing methodology #3 (Less time)	1.1	1.6	1.8	7.8
Toxicity‐testing methodology #4 (Less uncertainty)	14	14	26	30
Toxicity‐testing methodology #5 (Less cost, less time, less uncertainty)	0.19	0.20	0.37	0.42

**Table VI:** Reduction in the Average and Maximum Values of the Cost‐Effectiveness Ratio (CER) Associated with Reductions in Cost, Time, and Uncertainty

	Reduction in Average CER Across all Values of $\mu_{\mathrm{tox}}$		Reduction in CER for the Value of $\mu_{\mathrm{tox}}$ most Impacted by Uncertainty	
	Simple Decision	Complex Decision	Simple Decision	Complex Decision
Reduction due lower cost (Meth. #1/Meth. #2)	5.0	5.0	5.0	5.0
Reduction due shorter duration of testing (Meth. #1/Meth. #3)	14	14	14	14
Reduction due less uncertainty (Meth. #1/Meth. #4)	1.1	1.6	1.5^1^	4.6^1^
Reduction due lower cost, shorter duration, and less uncertainty (Meth. #1/Meth. #5)	80	111	108	327

^1^ Values are determined based on the chemical with the largest difference between toxicity‐testing methodologies #1 and #4. This chemical may not have the largest value of CER.

## 5 DISCUSSION

The focus of this article is the development of a framework for the selection of cost‐effective toxicity‐testing methodologies for the systematic testing of the large numbers of chemicals in commerce that have little or no toxicity data. The framework addresses the issue of how to perform tradeoffs between varying levels of uncertainty, cost, and duration by placing the three elements into a common metric, CER, that is quantitative, objective, and transparent. The framework can be adapted to incorporate the specific characteristics of different toxicity‐testing methodologies, the nature of the chemicals under consideration, the chemical and endpoint‐specific dose‐response models, and the various decision rules used by different regulatory programs.

The illustrative applications presented in the article are designed to explore the relative impacts of fivefold differences in costs, durations, and uncertainties of different toxicity‐testing methodologies. Results from these examples show that the reductions in all three elements provide benefits to decisionmakers. The relative changes in CER suggest that a fivefold change in cost consistently provides a fivefold benefit to decisionmakers by increasing the throughput of a testing program. The benefits of a fivefold reduction in duration varied with the duration of the time horizon. For shorter time horizons, the benefits are many times larger than those from reductions in cost. For longer time horizons, the benefits are smaller. Shorter time horizons are more appropriate for testing programs for groups of priority chemicals where there are immediate concerns about health risks. Longer time horizons are more appropriate for testing programs that systematically test chemicals of various levels of concern. It should be noted that cost and duration are likely to be positively correlated, since longer duration tests will require more resources and tie up laboratory space for longer periods of time than shorter duration tests. As a result, studies of a shorter duration are likely to have lower costs, resulting in larger reductions in CER. Using the assumptions in Table IV, a methodology that is fivefold lower in both cost and duration would result in a 71‐fold reduction in CER for both the simple and complex decisions.

The benefit of the fivefold reduction of $\sigma ({\hat{\mu }}_{\mathrm{tox}}^{j})$ used in the examples is more complex than the reductions in cost and duration. Like duration of testing, uncertainty in test results is not a direct input to the CER equation. Instead, the effects of uncertainty are mediated through changes in the DMV. The value of the reduction in the examples varies across chemicals since the likelihood of a given level of uncertainty in a toxicity finding resulting in an incorrect decision is a function of how close the risks posed by a chemical are to the TRL or a decision point. For the simple decision, the reduction of uncertainty reduced the CER by at most 1.5‐fold for chemicals with certain toxicities. The impact of the reduction in uncertainty is more important in the complex decision than the simple decision. The largest reduction in CER in the complex decision from the uncertainty reduction is 4.6‐fold. For many chemicals, including those that posed no risk, there is little or no reduction in CER with the reduction in uncertainty. These findings suggest that regulatory programs making simple screening decisions (are risks acceptable or unacceptable?) on large numbers of chemicals, and where the chemicals have a wide range of toxicities, may be better served by testing methodologies with shorter durations and lower costs, rather than longer and more expensive testing methodologies that result in greater reductions in uncertainty.

The findings of this article provide support for the concept of tiered approaches to testing of chemicals (Thomas et al., 2013; Gannon et al., 2019). Such approaches would perform an initial tier of testing using methods with short durations and low costs, but with uncertainties that may be higher than traditional testing. Following the initial testing, the risks posed by exposures to the chemicals would be assessed. If the assessment indicates that risks from existing exposures are well below the TRL, then the testing would stop, and corresponding chemical exposures would be defined as being of low concern. If the assessment indicated that the chemical could pose a risk that is greater than the TRL, then a second tier of testing would be performed. The methods used in the second tier would be selected to offer lower levels of uncertainty than the testing methods used in the first tier. Such tests are likely to have higher costs and may have longer durations. By reserving such tests for the second tier, the approach would increase the speed and decrease the costs of assessing risks from large numbers of chemicals.

Such a tiered approach may also use data on exposure levels in the selection of the testing methodology. Since risk is a function of both exposure and toxicity, chemicals with low exposures are more likely to offer lower risks than chemicals with high exposures. As a result, toxicity testing for the initial tier of low‐exposure chemicals could be performed using lower cost and shorter duration, but more uncertain, methodologies. Chemicals with high levels of exposure may warrant an initial tier of testing that uses less uncertain methods.

When defining the output for the CEA, the framework does not propose an “acceptable” level of accuracy for toxicity findings. Instead, the framework requires that the analyst to determine the probability that the toxicity estimate from a particular testing methodology will result in a correct decision. This approach acknowledges the fact that the acceptability of the uncertainty in a toxicity estimate is a function of how the estimate is used in risk‐based decision making. Requiring a fixed level of accuracy for a toxicity finding without considering how the finding will be used would be an essentially arbitrary decision.

While it is more appropriate to evaluate the ability of a methodology in terms of the ability of the methodology's results to support a specific type of decision, such a finding will vary with the nature of the decision. A methodology that works well for a simple decision‐making process may not be sufficiently accurate for a more‐complex decision. As a result, values of CER will differ for different decisions potentially resulting in a different choice of testing methodology. The ability to evaluate the value of data for a specific decision also suggests that the framework could provide a quantitative basis for a determination of whether toxicity findings are adequate to support a specific decision. This would provide an objective measure of whether a testing methodology is “fit‐for‐purpose” for a specific decision (Meek et al., 2013).

The definition of the CER is based on the cost of a correct decision as defined by Equation (7) and does not consider the number of incorrect decisions the testing methodology might also generate. In the examples above, a methodology that would generate 20 correct decisions and 50 incorrect decisions would have a higher CER than a methodology which generates 19 correct decisions and no incorrect decisions (when costs and durations are similar). This suggests that the DMV (a measure of the probability of a correct decision) might be used to determine if a methodology would generate large numbers of incorrect decisions for a specific decision‐making rule and, if so, it may not be “fit for purpose.” The framework also does not discriminate between errors that lead to over‐regulation and errors that lead to under‐regulation. Future versions of the framework can include the use of uncertainty factors (U.S. EPA, 1991) for toxicity predictions that can reduce the chance of under‐regulation.

As a demonstration of the framework, illustrative examples are developed using a postulated, but plausible, risk model (wherein toxicity and exposure distributions are assumed to follow lognormal distributions), two specific risk‐based decision‐making approaches (SDR and CDR), and five hypothetical toxicity‐testing methodologies. These examples demonstrate how the framework addresses a simple and a more complex decision; however, the examples do not fully explore the range of risk‐based decision‐making approaches that might be encountered in practice, and additional work in this area is needed. In addition, only two values for cost, duration, and uncertainty are modeled. Future work could examine the impact of these elements in greater detail and over a wider range of values.

Finally, the framework can be extended to consider uncertainty in the variation of toxicity across individuals and the uncertainty in estimates of the mean and variation in exposure across individuals. For example, the use of probabilistic techniques to characterize risk allows the consideration of multiples sources of uncertainty in toxicity and exposure data and does not require the assumption of lognormality in inter‐individual variation in dose and toxicity.

In summary, the framework presented here represents an attempt to use CEA to address the issue of selecting a toxicity‐testing methodology that can be applied to the large numbers of untested chemicals in commerce. Two example CEAs created using the framework found that the three elements (cost, duration, and uncertainty) are all important considerations in selection of a testing methodology. Under the specific assumptions underlying the analyses conducted, reductions in testing duration and testing costs are more important than reductions in uncertainty in toxicity findings.

## APPENDIX 1

### DERIVATION OF AN ANALYTICAL SOLUTION FOR PREDICTING POPULATION RISK

In section 2.3 of this article, we assert that there is an analytical solution (Equation (3) of the text) to the integral of toxicity and exposure distributions that defines population risk (Equation (2) of the text). Specifically, we state that:

(A.1)
$$\begin{array}{ccc} R & = & \int G_{\mathrm{tox}}\left(x|\theta_{\mathrm{tox}}\right)f_{\exp}\left(x|\theta_{\exp}\right)\begin{array}{ccc} \mathrm{dx} & = & \Phi \left(\frac{\mu_{\exp}-\mu_{\mathrm{tox}}}{\sqrt{\sigma_{\exp}^{2}+\sigma_{\mathrm{tox}}^{2}}}\right) \end{array} \end{array}$$

The following is a proof for this assertion.

The assumption is made in section 2.3 that we have $X|\theta_{\mathrm{tox}}∼LN\left(\mu_{\mathrm{tox}},\sigma_{\mathrm{tox}}\right)$ and $X|\theta_{\exp}∼LN\left(\mu_{\exp},\sigma_{\exp}\right)$. Let Y=log(X), then we have $Y|\theta_{\mathrm{tox}}∼N\left(\mu_{\mathrm{tox}},\sigma_{\mathrm{tox}}\right)$ and $Y|\theta_{\exp}∼N\left(\mu_{\exp},\sigma_{\exp}\right)$. Let $Z=\frac{Y-\mu_{\exp}}{\sigma_{\exp}}$, then Z∼N(0,1) (i.e., Z follows standard normal distribution). This means that $Y=\sigma_{\exp}Z+\mu_{\exp}$.

Let Φ(·) denote the CDF for standard normal distribution, and $f_{z}$ be the pdf of standard normal distribution. We can express $G_{\mathrm{tox}}\left(x\mu_{\mathrm{tox}},\sigma_{\mathrm{tox}}\right)$ as a function of z as:

(A.2)
$$\begin{array}{ccc} G_{\mathrm{tox}}\left(x\mu_{\mathrm{tox}},\sigma_{\mathrm{tox}}\right) & = & \Phi \left(\frac{y-\mu_{\mathrm{tox}}}{\sigma_{\mathrm{tox}}}\right)\begin{array}{cc} = & \Phi \left(\frac{\sigma_{\exp}z+\mu_{\exp}-\mu_{\mathrm{tox}}}{\sigma_{\mathrm{tox}}}\right)\begin{array}{cc} = & \Phi \left(\frac{\left(\mu_{\exp}-\mu_{\mathrm{tox}}\right)}{\sigma_{\mathrm{tox}}}+\left(\frac{\sigma_{\exp}}{\sigma_{\mathrm{tox}}}\right)z\right). \end{array} \end{array} \end{array}$$

Then we have:

(A.3)
$$\begin{array}{ccc} & & \int G_{\mathrm{tox}}\left(x|\theta_{\mathrm{tox}}\right)f_{\exp}\left(x|\theta_{\exp}\right)\mathrm{dx}=\int \Phi \left(\frac{\left(\mu_{\exp}-\mu_{\mathrm{tox}}\right)}{\sigma_{\mathrm{tox}}}+\left(\frac{\sigma_{\exp}}{\sigma_{\mathrm{tox}}}\right)z\right)f_{z}\left(z\right)\mathrm{dz} \end{array}$$

from Owen (1980), we have:

(A.4)
$$\int \Phi \left(a+bz\right)f_{z}\left(z\right)dz=\Phi \left(\frac{a}{\sqrt{1+b^{2}}}\right)$$

Defining a as $\frac{\left(\mu_{\exp}-\mu_{\mathrm{tox}}\right)}{\sigma_{\mathrm{tox}}}$ and b as $\frac{\sigma_{\exp}}{\sigma_{\mathrm{tox}}}$, Equation (3) is shown to be the solution of the integral.

(A.5)
$$\begin{array}{ccc} \Phi \left(\frac{a}{\sqrt{1+b^{2}}}\right) & = & \Phi \left(\frac{\frac{\left(\mu_{\exp}-\mu_{\mathrm{tox}}\right)}{\sigma_{\mathrm{tox}}}\;}{\sqrt{1+{\left(\frac{\sigma_{\exp}}{\sigma_{\mathrm{tox}}}\right)}^{2}}}\right)\\ & = & \Phi \left(\frac{\mu_{\exp}-\mu_{\mathrm{tox}}}{\sqrt{{\left(\sigma_{\mathrm{tox}}\right)}^{2}+{\left(\sigma_{\exp}\right)}^{2}}}\right) \end{array}$$

## APPENDIX 2

### IMPACT OF THE SELECTION OF A TIME HORIZON (TH) ON THE RELATIONSHIP BETWEEN DURATION OF TOXICITY TESTING AND CER

Sections 2.2 and 3.1 present the calculation of CER and the inputs for that equation used in the example CEAs (Equation (1)). As the text indicates, the output used in determining the value of CER is a function of the number of years when toxicity data are available to support risk‐based decisions and when in the future this period of availability occurs. Larger durations for toxicity testing and analysis reduce the value of the CERby delaying the time when testing results are available, reducing the number of tests that can be completed during the time horizon, and reducing the number of years that the test results are available in the time horizon.CER is also a function of the timing of testing costs, with delayed costs being favored. Finally, larger discount rates increase the impact of longer testing durations.

The factor that has the largest impact, however, is the selection of the value of THused in the CEA. Short THs disproportionally penalize the toxicity‐testing methodologies with longer durations. Table AI presents an assessment of the values of CER for toxicity‐testing methodologies #1 and #3. These methodologies differ only by the duration of the test (two years and ten years respectively). With one exception, this calculation uses the same assumptions as toxicity‐testing methodologies #1 and #3 presented in the text of this article (i.e., the value ofDMV for both methodologies are assumed to be 1 for the chemical). The last three columns of the table present the CER values for toxicity‐testing methodologies #1 and #3 and the ratios of these two CER values for THs ranging from 11 to 20 years. The ratio of CER values for the two methodologies is 49 for a TH of 11 years and declines to a value of 3.4 for a TH of 20 years.

The reason for the decline is that for a time horizon of 13 years, toxicity‐testing methodology #1 has only one year of data available for two chemicals, but at 13 years toxicity‐testing methodology #3 has data on 18 chemicals and some of the chemicals have had data available for as long as 8 years. For a TH of 20 years, both methodologies lead to an increase in the number of chemicals tested and the average length of time the data are available, but the fractional increase is much larger for the toxicity‐testing methodology #1 than #3.

**Table AI:** Impact of the selection of a duration for time horizon on the relationship between the duration of toxicity testing and the cost‐effectiveness ratio (CER)

Time Horizon	Budget	Toxicity‐Testing Methodology #1 Test Duration of 10 Years		Toxicity‐Testing Methodology #3 Test Duration of 2 Years		CER for Different Durations of the Time Horizon		
Year	Annual Costs (Net Present Value (Millions))	Number of Chemicals with Results	Discounted Value of the Outcomes (Years)	Number of Chemicals with Results	Discounted Value of the Outcomes (Years)	Toxicity‐Testing Method. #1 Test Duration = 10 years	Toxicity‐Testing Method. #3 Test Duratio*n* = 2 years	Ratio of CER Values
1	$10	0	0	0	0	‐	‐	‐
2	$9.7	0	0	0	0	‐	‐	‐
3	$9.4	0	0	2	1.9	‐	$15.45	‐
4	$9.2	0	0	4	3.7	‐	$6.90	‐
5	$8.9	0	0	6	5.3	‐	$4.34	‐
6	$8.6	0	0	8	6.9	‐	$3.14	‐
7	$8.4	0	0	10	8.4	‐	$2.45	‐
8	$8.1	0	0	12	9.8	‐	$2.01	‐
9	$7.9	0	0	14	11.1	‐	$1.71	‐
10	$7.7	0	0	16	12.3	‐	$1.48	‐
11	$7.4	2	1.5	18	13.4	$64.04	$1.31	49
12	$7.2	4	2.9	20	14.4	$23.42	$1.18	20
13	$7.0	6	4.2	22	15.4	$12.76	$1.07	12
14	$6.8	8	5.4	24	16.3	$8.29	$0.98	8.5
15	$6.6	10	6.6	26	17.2	$5.96	$0.90	6.6
16	$6.4	12	7.7	28	18.0	$4.56	$0.84	5.4
17	$6.2	14	8.7	30	18.7	$3.66	$0.79	4.7
18	$6.1	16	9.7	32	19.4	$3.03	$0.74	4.1
19	$5.9	18	10.6	34	20.0	$2.57	$0.70	3.7
20	$5.7	20	11.4	36	20.5	$2.23	$0.66	3.4
